# Rare, rarer, it still has not happened

**DOI:** 10.3325/cmj.2019.60.565

**Published:** 2019-12

**Authors:** Branimir K. Hackenberger

**Affiliations:** Department of Biology, Josip Juraj Strossmayer University, Osijek, Croatia *hackenberger@biologija.unios.hr*

There is an immense number of problems associated with rare diseases. In addition to the difficulties of posing a reliable diagnosis and administering proper treatment, several issues are stemming from the fact that a disease is rare. Depending on whether it is a rare infectious or a rare autoimmune disease, the problems and questions that need to be answered also differ. In the case of rare infectious diseases, we are often interested in the likelihood of its occurrence in an area, its estimated mortality, and other epidemiology-related issues. When it comes to rare autoimmune diseases, one may be interested in the extent to which they are present in a population or the survival period after the onset of the first symptoms. Good knowledge of rare diseases is critical, and their successful management is an interdisciplinary and transdisciplinary problem. Another term for rare diseases is “orphan diseases.” This name has almost nothing to do with medicine, but it has to do with economic science. Namely, when rare diseases are concerned, the market usually lacks drugs and other supplies for their diagnosis and treatment. The reason for this lies precisely in the small number of patients and the unprofitable production and storage of medicines and other necessities. On the other hand, some rare diseases are more common in some countries and less common in others. Moreover, in some countries, some rare diseases are actually common. In any case, it is becoming increasingly important to predict the number of patients and other quantitative facts related to rare diseases, including the economic consequences. However, an excellent prediction is not possible without a quantitative approach and methods. But how to handle data that are scarce, deficient, or practically nonexistent? These problems are addressed by rare event statistics.

A big problem in understanding some of the computational procedures is that they are not intuitive. The purpose of this column is to make the basic contemporary statistical ideas, techniques, and ways of thinking understandable to biomedical scientists and professionals. Therefore, this text will attempt to explain how the probabilities of rare events or events that have never occurred can be calculated.

**Figure 1:**
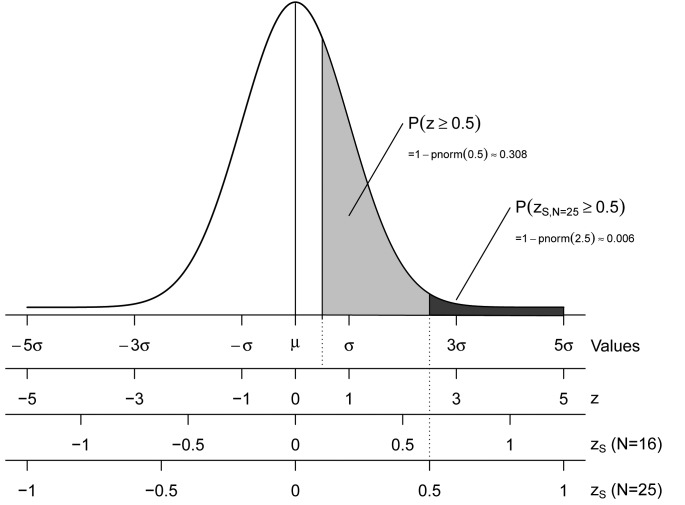
Relation of sample z-scores to population z-scores in the standard normal distribution.

**Figure 2:**
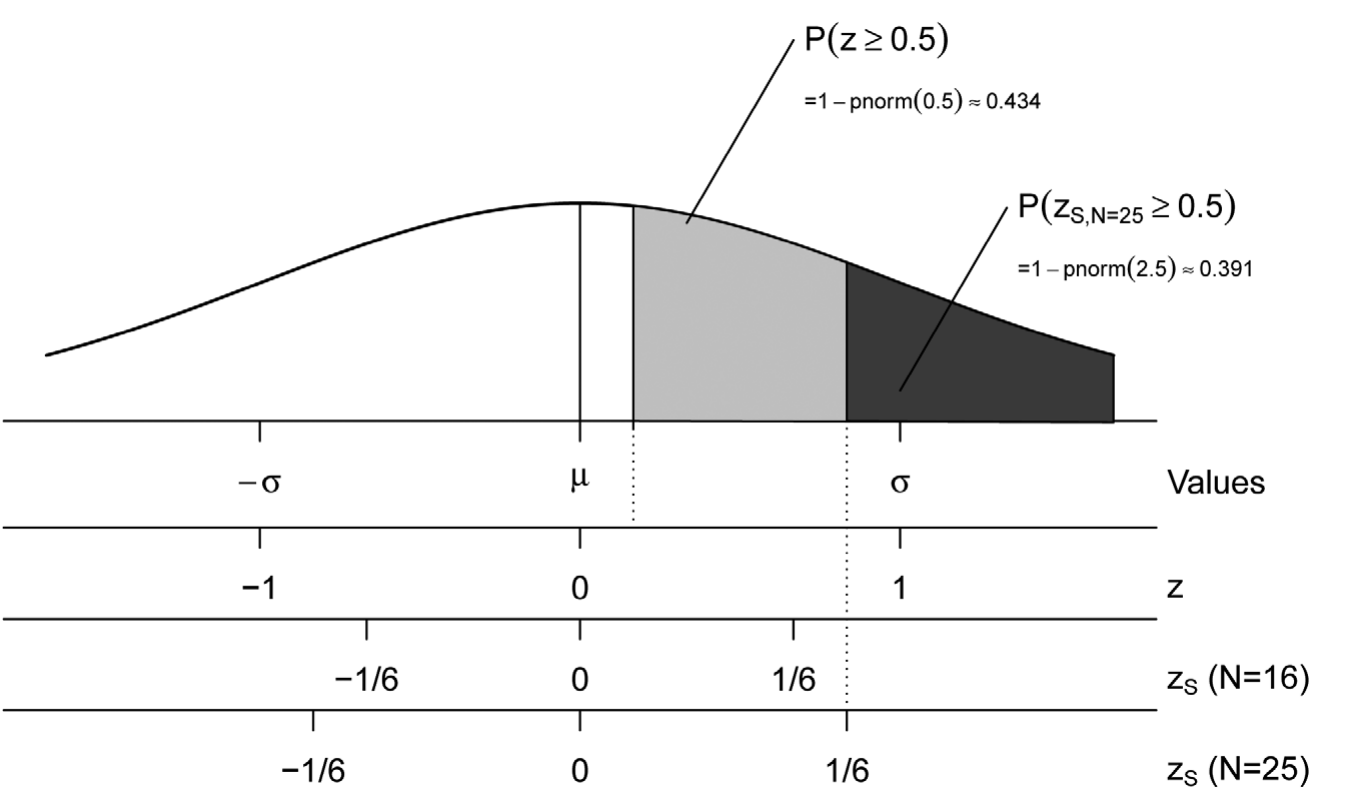
Relation of sample z-scores to population z scores in the normal distribution, with μ = 0 and σ = 3.

First of all, let us say something about the term “rare event” within conventional frequentist statistics. Take, for example, some medical parameter, X, with a mean value, μ, and standard deviation, σ, 10% of μ. What is the probability that a sample of size N = 25 will have a mean that is 5% greater than μ or greater? We will intuitively conclude that such a probability is high. Namely, if we know that the observed size is normally distributed then we can easily conclude, and calculate using the z -score, that the probability *P* = 0.31, ie, 31%. Wrong! The standard deviation of the sample is not equal to the standard deviation of the population. In other words, this result refers to the probability of the occurrence of an X that is 5% greater than μ or greater, rather than the probability of a sample mean. As our sample size is N = 25, the standard deviation of the sample is the standard deviation of the population divided by the root of the sample size, ie, 10/4 = 2.5% of the mean of the population, eg, μ. Therefore, the z-score of the sample mean is not 0.5 (50% of population standard deviation) but 5 / 2.5 = 2 (200% of population standard deviation). It is easy to calculate that the probability of such a value or a greater one is *P* = 0.0062, ie, 0.62%. Therefore, it is very small (Figure 1). What does this mean? If a sample of size N = 25 is taken from the population with the above assumptions, and the mean value of that sample is equal to or greater than the mean value increased by 5%, it can mean two things: 1) a rare event has occurred or 2) our mean value assumption and/or standard deviation is incorrect, ie, the true population mean is higher than assumed in this case. As a rule of thumb, we most often accept the second conclusion and decide that the true mean is most likely greater than the assumed one. As can be seen from this example, the sample size determines the probability limit below which an event will be considered rare, ie, unlikely. The smaller the sample, the higher the deviation of the sample mean from the assumed population mean. If, in this example, 125 samples were taken instead of 25, the probability that the mean of such a sample would deviate from the assumed mean value would be almost zero (2.87 × 10^−7^). But, what if the assumed standard deviation is 30% and not 10% as in the example? In this case, it is easy to calculate that the probability of the mean value of the sample with the given deviation is about 4% (Figure 2). In such conditions, a rare event would not be considered, and the assumptions about population parameters would not be questioned. But how do you predict and determine the likelihood that a rare event will occur? This is not difficult if we know the parameters of the population from which the sample is taken. But what if we have very little information?

Let us suppose we observed patients arriving at a doctor’s office for one day. The first ten patients had blood pressure problems. They were followed by ten patients with the flu and ten patients with a common cold. Based on these data, in what way could a model be used to predict the type of patient to come next? Let us call the patients A, B, and C, depending on the kind of problem they have. From these initial data, it can easily be concluded that after a patient with one type of a problem, a patient with the same problem is most likely to come next. This did not happen just two times during that day. Specifically, after a patient with blood pressure there came a patient with the flu, and after a patient with the flu there came a patient with a common cold. A simple model based on these initial data would deem the possibility that a patient C would come after a patient A or that a patient A would come after a patient C impossible. We know from experience that this is meaningless and that the information we measured is random. On the other hand, we know that we have very little data available to make a good prediction model.

**Figure 3:**
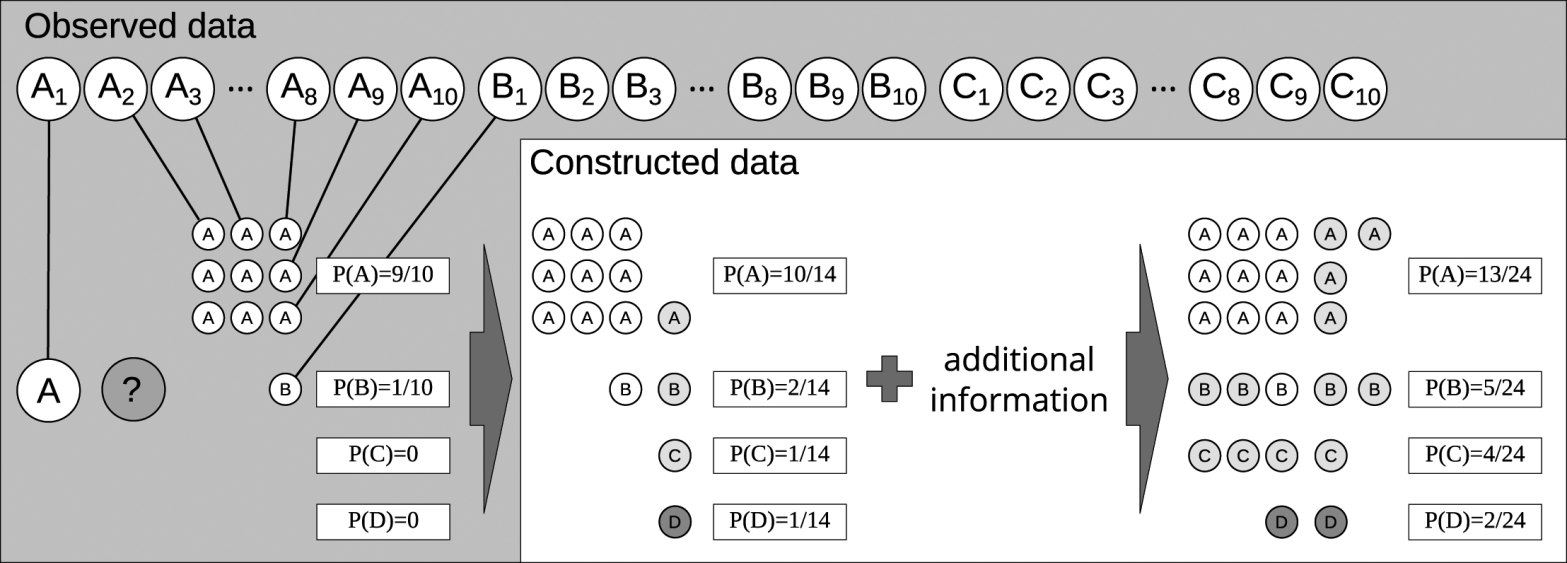
Simplified presentation of data “smoothing” with the introduction of a never occurred event.

Now, let us suppose that the first patient on the next day is patient A. The probability that the next patient will also be A is 9/10, the probability that it will be B is 1/9, and the probability that it will be C is zero. However, we know that in addition to these three types of patients, we can expect a patient D, who was not present on the day the study was performed. According to the existing research data, the likelihood that a patient D will arrive is zero because such a patient has never come, and is not included in the model. Just as we realistically understand that the probability that a patient A will come after a patient A is greater than zero, as is the probability that a patient will have symptoms we have not yet met, such as those of a rare disease. The simplest method to incorporate chance into a model based on experience, expectations, or prior knowledge is the so-called “add-one smoothing method.” For this purpose, we will fabricate the data for the event that the next patient will be C, as well as for the event that the next patient will be D, in such a way that we will give each event an additional chance (Figure 3). That is, we will insert four dummy events into the existing data. So now, the probability that the next patient will be A is 10/14, that it will be B is 2/14, that it will be C is 1/14, and that it will be D is 1/14. Are the data modified like this completely fake? Well, not really. Namely, as I wrote earlier, we know that the model made according to the obtained data are meaningless. It is meaningless because we are aware that the next patient may be any of the three known types (A, B, and C) and one unknown (D). We assume that all four equally share the probability, that is, each of them carry 1/4 of the added probability, that is, each event is added to one type of patient.

According to such “fabricated” data, the likelihood of a patient C or D coming after A is not zero. Furthermore, let us suppose we have data on the proportion of individual types of patients in the area where the doctor’s office is located so that we know *a priori* that about 30% are A type patients, 30% are B type patients, 30% are C type patients, and about 10% of patients have never visited the doctor. Therefore, ten more events should now be added, three for patients A, B, and C and one for patients D, so the probabilities will now be 13/24 for A, 5/24 for B, 4/24 for C, and 2/24 for D.

**Figure 4:**
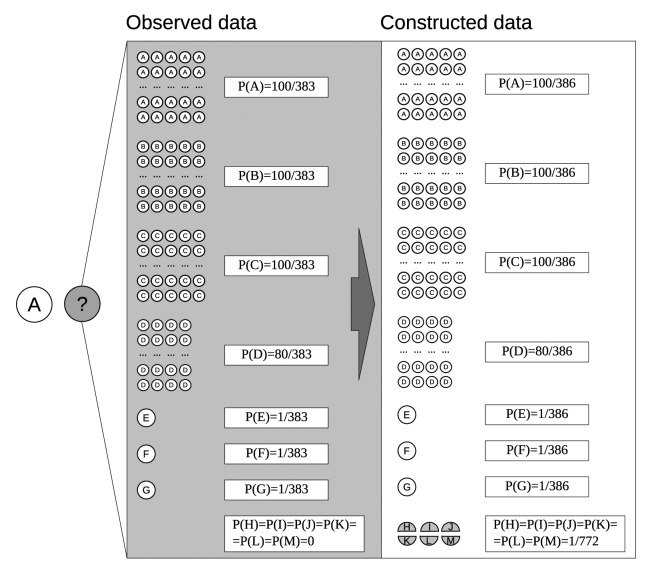
Simplified presentation of the Good-Turing estimate method with the introduction of six never occurred events.

The Good-Turing estimate method is somewhat better (Figure 4). Let us suppose we have slightly more sophisticated information on the sequence of patient arrivals so that we know that after a patient A, a patient A came 100 times, patient B 100 times, patient C 100 times, patient D 80 times, patient E once, patient F once, and patient G once. It has never happened that after a patient A came patients H, I, J, K, L, M, or N, which we know or expect to exist. As we are aware that it is not impossible for a patient of any type to arrive after a patient A, ie, as we know *a priori* that this is logically possible, we will consider that the number of events that never happened is equal to the number of events that occurred only once. In our example, only three events occurred once. As there are six rare events that never happened, each will receive 3/6 events. In other words, the number of occurrences of any of the six rare events is 1/2, and the total number of events is increased by 3.

Both described methods are the basis for a maximum *a posterior* probability estimate (MAP). MAP is a term from Bayesian statistics, and the distributions we constructed in the previous two examples are “prior” distributions as the beginning of further computation. The prognosis and calculation of parameters of rare events, ie, events that have not yet occurred, are becoming increasingly successful with the application of Bayesian statistics within the development of machine learning and AI algorithms.

Researchers dealing with rare diseases are increasingly applying methods of Bayesian statistics and machine learning. Thus, Taroni et al (1), because of the extremely low number of rare disease cases reported compared with common chronic diseases, collected data from several studies with different experimental settings, tissues, and biological conditions. The database created was a pathway-level information extractor (PLIER) model. The predictive model was tested on larger data sets and, as expected, the detection of rare diseases has improved compared with models trained on smaller data sets. Kong et al (2) applied computer vision and machine learning to early detect acromegaly, while Fabregat et al (3) automatically filtered information on the phenotypic features and disabilities resulting from rare diseases using deep neural networks. The results of the model (disease-disability pairs) are essential for post-detection of rare diseases and can prepare patients for difficulties they may encounter in the future. Fujiwara et al (4), using text-mining techniques (co-occurrence), created databases of phenotypic characteristics associated with rare diseases. Garcelon et al (5) developed a model to automate the finding of similarities between new patients and patients with established diseases. The rare diseases they focused on were Lowe syndrome, dystrophic epidermolysis bullosa, activated PI3K delta syndrome, Rett syndrome, and Dowling Meara. MacLeod et al (6) attempted to determine the behaviors and habits of people with common chronic illnesses and those with rare diseases based on survey results.

Although electronic health records contain information about rare diseases, designing models based on such databases is difficult because of the small number or lack of a gold standard of patients and associated symptoms. Colbaugh et al (7) developed an algorithm based on supervised ensemble learning and unsupervised clustering that provides robust, noise-based learning. The trained model accurately predicted the incidence of rare diseases and allowed the post-diagnosis of patients enrolled in an electronic medical database. Particularly impressive is the work of Alirezaie et al (8), who developed a predictive model based on machine learning to determine the genetic disease determinants. The system was tested on a data set from databases that stored the genotypes of patients with cancer and rare diseases.

The above examples and a few recent works show that computer techniques based on Bayes statistics and machine learning and high-performance calculations have become indispensable in situations of both lack of sufficient data and for any meaningful prognostication of rare events and events that have not (yet) occurred.
